# Supplementary material for: Curing Behavior, Rheological, and Thermal Properties of DGEBA Modified with Synthesized BPA/PEG Hyperbranched Epoxy after Their Photo-Initiated Cationic Polymerization
Source: Polymers (Basel). 2020 Sep 29;12(10):2240. doi: 10.3390/polym12102240 (PMC7599708; doi:10.3390/polym12102240)
Supplement: Supplementary file 1 [file polymers-12-02240-s001.pdf]

polymers

MDPI

Article

Curing Behavior, Rheological, and Thermal Properties of DGEBA Modified with Synthesized BPA/PEG Hyperbranched Epoxy after Their Photo-Initiated Cationic Polymerization

Tossapol Boonlert-uthai [1], Kentaro Taki [2] and Anongnat Somwangthanaroj [1,*]

[1] Department of Chemical Engineering, Faculty of Engineering, Chulalongkorn University, Bangkok 10330, Thailand; tossapol.bu@gmail.com

[2] School of Mechanical Engineering, Kanazawa University, Kanazawa 920-1192, Japan; taki@se.kanazawa-u.ac.jp

* Correspondence: anongnat.S@chula.ac.th; Tel.: +66-2218-6860

Supplementary Material

Table 1. The density of each sample cured at various UV intensity.

UV intensity (mW/cm^2)	Density (g/cm^3)	
	DGEBA	D90H10
10	1.0000 ± 2×10^{-6}	1.0000 ± 1×10^{-5}
20	1.0000 ± 9×10^{-8}	1.0000 ± 5×10^{-6}
30	1.0000 ± 1×10^{-5}	1.0000 ± 5×10^{-6}
40	1.0000 ± 1×10^{-5}	1.0000 ± 5×10^{-6}
50	1.0002 ± 4×10^{-4}	1.0002 ± 3×10^{-4}

Table 2. Parameters from Zimm plot for DGEBA and D90H10 at various UV intensity.

UV intensity (mW/cm^2)	Slope	Intercept	I_0	ξ (nm)	R_g (nm)
DGEBA					
10	55.547 ± 0.440	0.009 ± 0.000	108.042 ± 1.349	7.747 ± 0.079	13.42 ± 0.14
20	54.391 ± 0.004	0.021 ± 0.000	48.567 ± 0.000	5.140 ± 0.000	8.90 ± 0.00
30	39.715 ± 0.063	0.027 ± 0.000	37.290 ± 0.021	3.848 ± 0.004	6.67 ± 0.01
40	45.547 ± 0.344	0.024 ± 0.000	41.289 ± 0.148	4.337 ± 0.024	7.51 ± 0.04
50	45.315 ± 0.003	0.024 ± 0.000	40.866 ± 0.000	4.303 ± 0.000	7.45 ± 0.00
D90H10					
10	52.440 ± 0.004	0.022 ± 0.000	44.783 ± 0.000	4.846 ± 0.000	8.39 ± 0.00
20	54.257 ± 0.004	0.020 ± 0.000	50.454 ± 0.000	5.232 ± 0.000	9.06 ± 0.00
30	66.274 ± 1.067	0.006 ± 0.000	176.712 ± 9.418	10.821 ± 0.377	18.74 ± 0.65
40	60.648 ± 0.004	0.016 ± 0.000	64.350 ± 0.000	6.247 ± 0.000	10.82 ± 0.00
50	58.156 ± 0.005	0.023 ± 0.000	44.131 ± 0.000	5.066 ± 0.000	8.77 ± 0.00

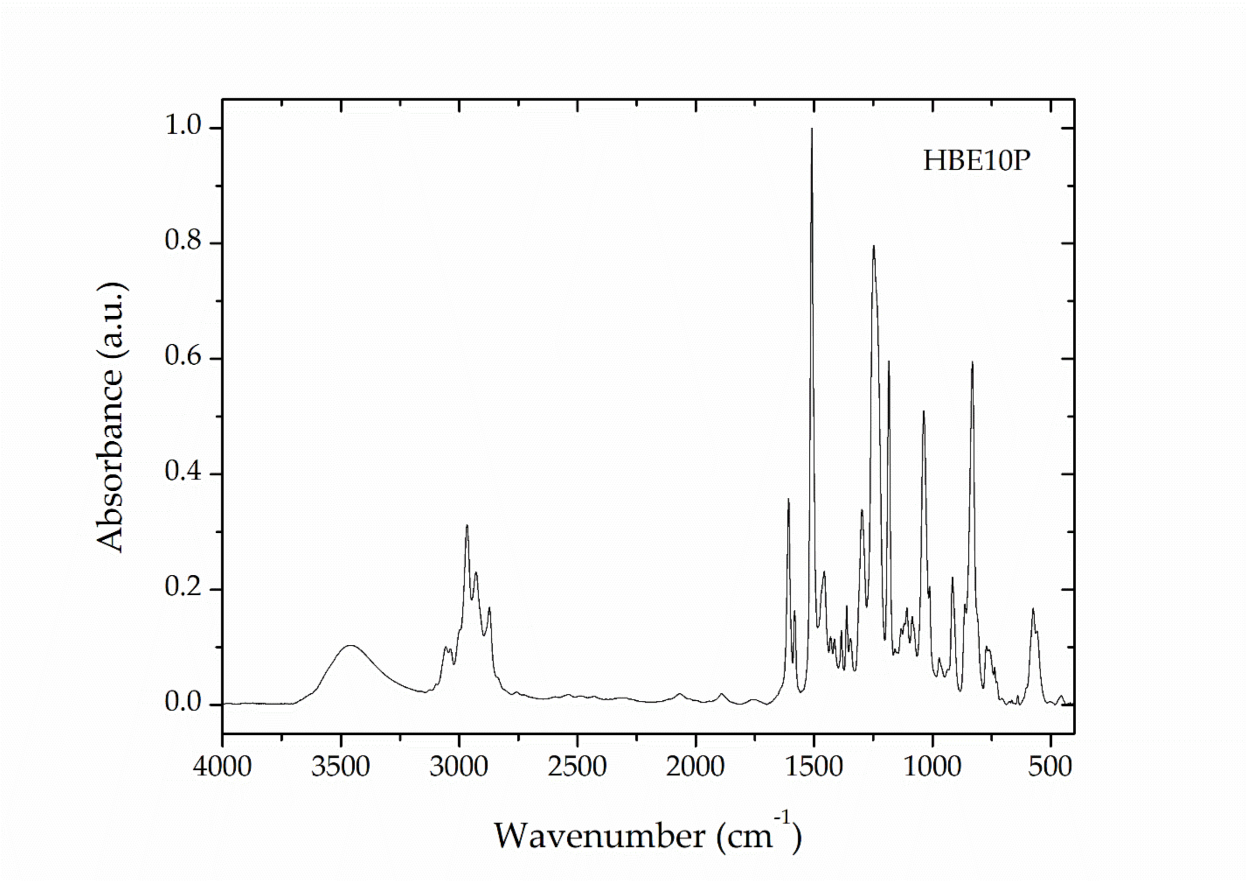

Figure 1. FTIR spectra of HBE10P resin.

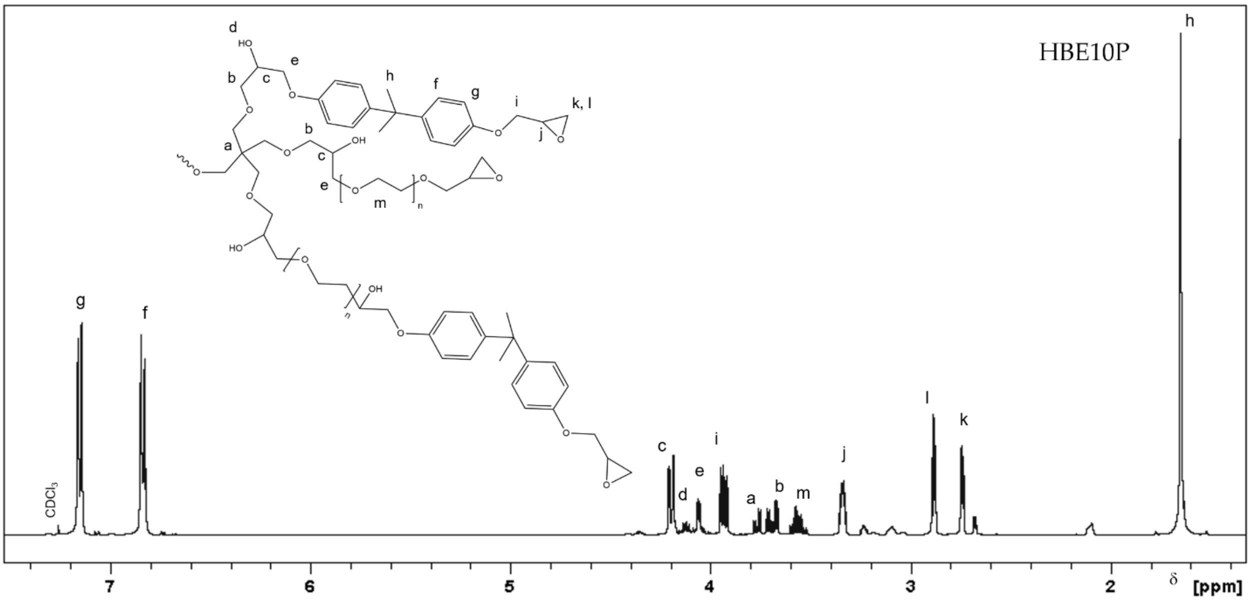

Figure 2. ^{1}H NMR spectrum of HBE10P resin.

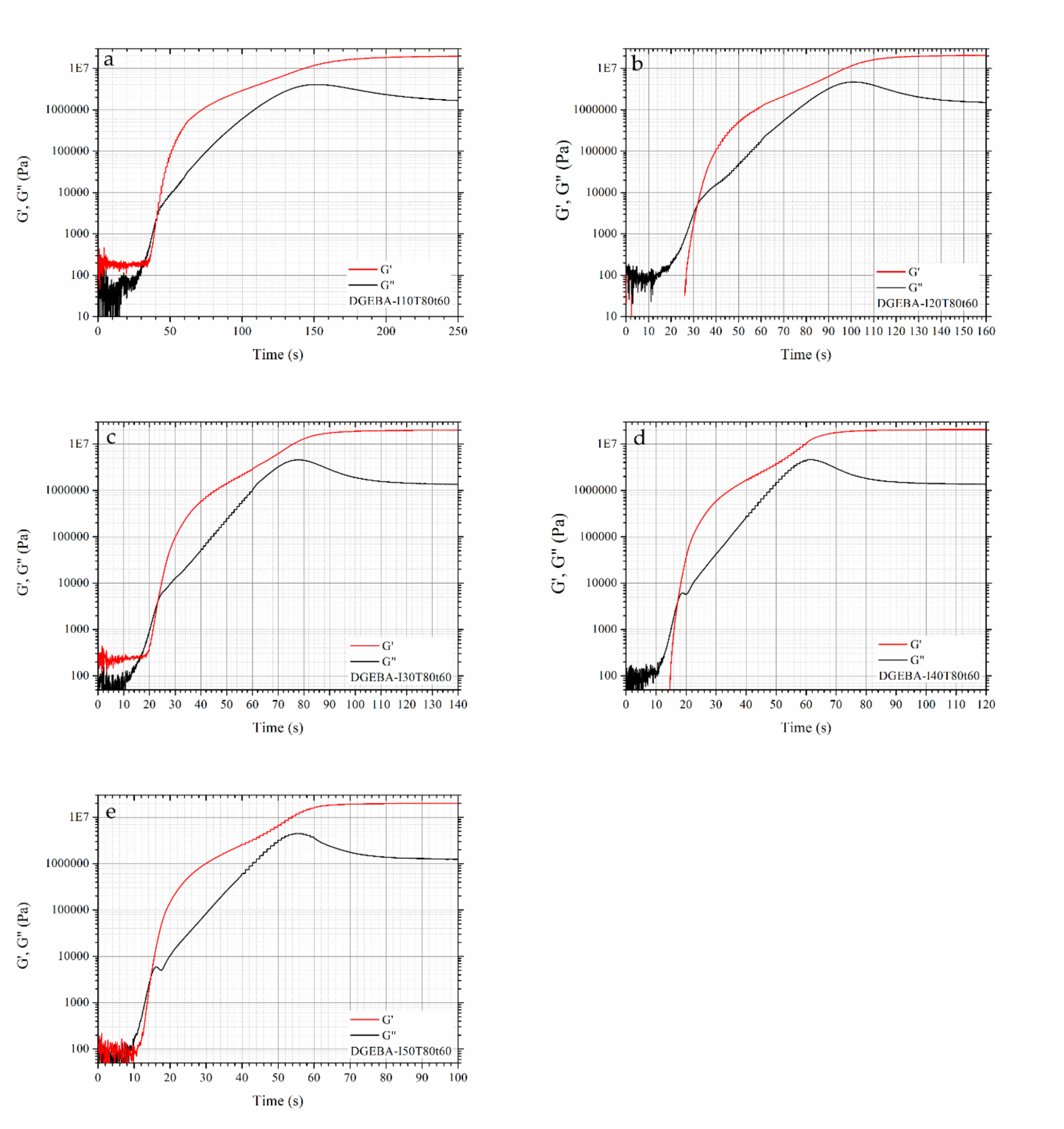

Figure 3. Crossover of storage modulus (G′) and loss modulus (G″) of DGEBA system at various curing conditions: (**a**) 10 mW/cm^2 (**b**) 20 mW/cm^2 (**c**) 30 mW/cm^2 (**d**) 40 mW/cm^2 and (**e**) 50 mW/cm^2.

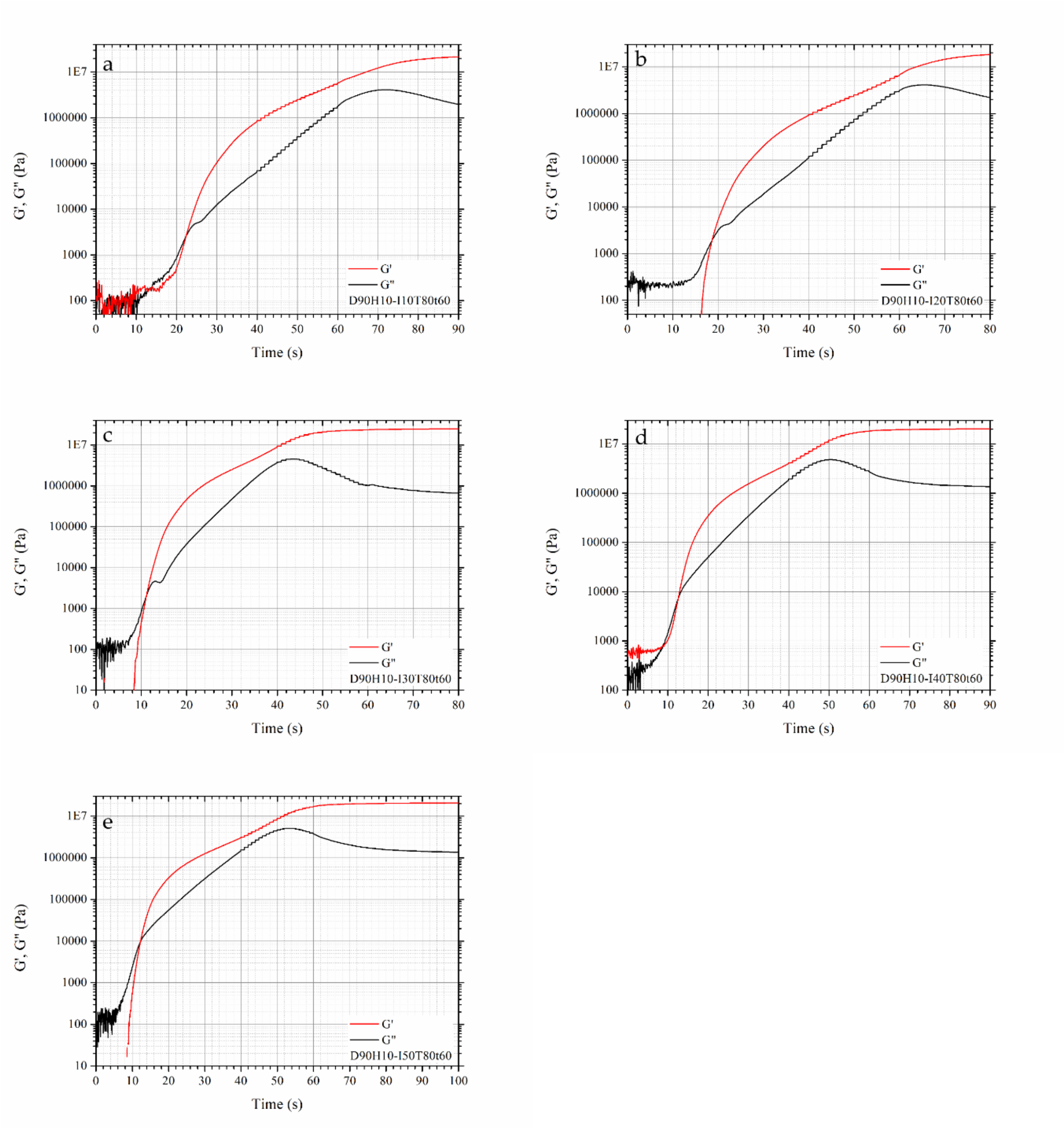

Figure 4. Crossover of storage modulus (G′) and loss modulus (G″) of D90H10 system at various curing conditions: (**a**) 10 mW/cm^2 (**b**) 20 mW/cm^2 (**c**) 30 mW/cm^2 (**d**) 40 mW/cm^2 and (**e**) 50 mW/cm^2.

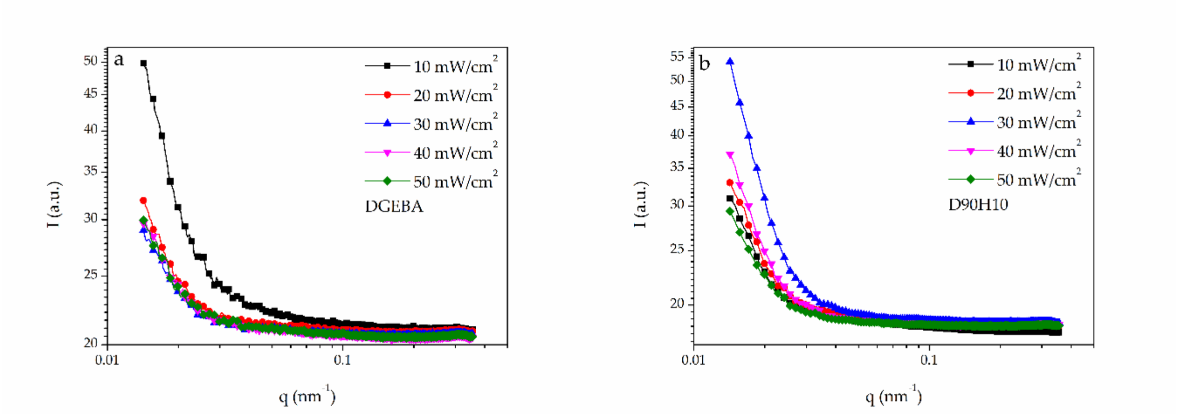

Figure 5. SAXS profiles of (**a**) DGEBA and (**b**) D90H10 cured at various UV intensity.

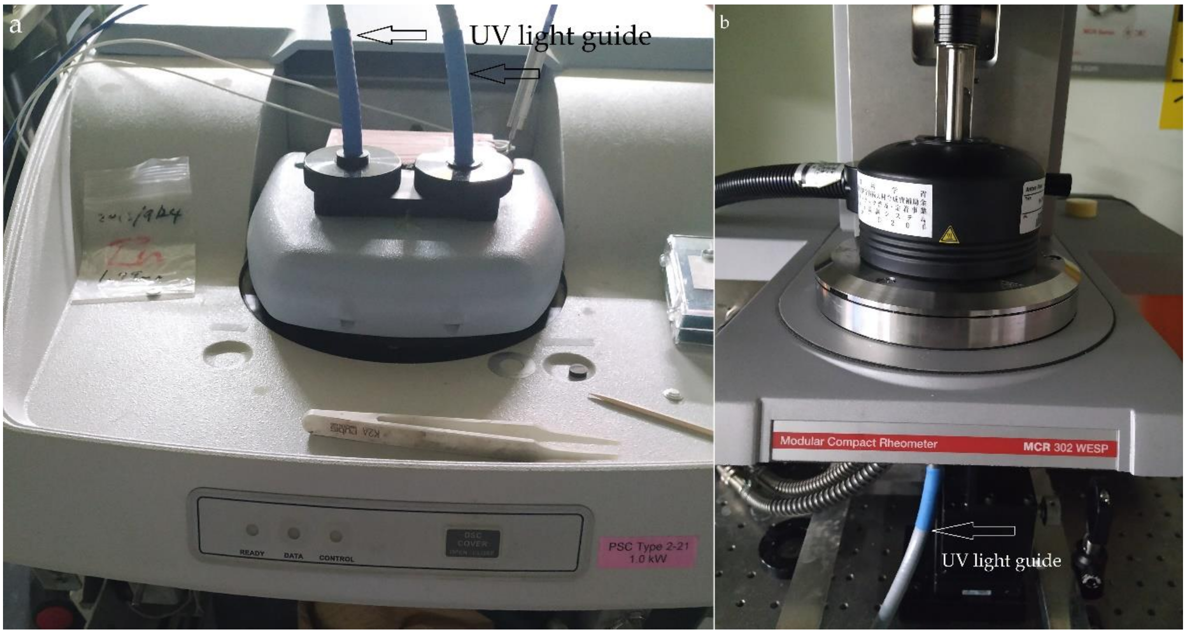

Figure 6. (**a**) DSC and (**b**) Rheometer instruments equipped with UV light guide.

Abbreviations

DGEBA	Diglycidyl ether of bisphenol A
BPA	Bisphenol A
PEG	Polyethylene glycol
HBE10P	Hyperbranched epoxy resin consisting of 10 wt% PEG
D90H10	Resin having DGEBA and HBE10P ratio to 90:10 wt/wt
ACE	Activated chain end mechanism
AM	Activated monomer mechanism
DSC	Differential scanning calorimetry
UV	Ultraviolet
SAXS	Small-angle X-ray scattering (SAXS)
RB	Round-bottom flask having a spherical bottom
